# PILRA is associated with immune cells infiltration in atrial fibrillation based on bioinformatics and experiment validation

**DOI:** 10.3389/fcvm.2023.1082015

**Published:** 2023-06-16

**Authors:** Weihua Shi, Xiaoli Li, Yongxing Su, Dezhao Liu, Liying Wu, Shuo Li, Wenxiu He, Guoqiang Zhong, Zhiyuan Jiang

**Affiliations:** ^1^ Department of Cardiology, First Affiliated Hospital, Guangxi Medical University, Nanning, China; ^2^ Department of Pharmacy, Guangxi Zhuang Autonomous Region People’s Hospital, Nanning, China

**Keywords:** atrial fibrillation, inflammation, immune cells 1nfiltration, weighted gene co-expression network analysis (WGCNA), least absolute shrinkage selection operator (LASSO) regression analysis

## Abstract

**Background and aims:**

inflammation plays an important role in atrial fibrillation (AF). In this study, we investigated the significance of immune cell infiltration in AF and identified the potential Hub genes involved in the regulation of immune cell infiltration in AF.

**Methods:**

we obtained AF datasets from the GEO database and analyzed them for obtaining differentially expressed genes (DEGs) by R software. Then, we performed GO, KEGG, and GSEA enrichment analyses of DEGs. The Hub genes of AF were determined by least absolute shrinkage selection operator (LASSO) regression analysis and weighted gene co-expression network analysis (WGCNA). Their validation was verified by using quantitative polymerase chain reaction (qPCR) in the AF rat model. Finally, we used a single sample GSEA (ssGSEA) to analyze immune cell infiltration and its relationship with hub genes.

**Results:**

We obtained 298 DGEs from the heatmap and found that DGEs were closely related to inflammation, immunity, and cytokine interactions by enrichment analyses. We obtained 10 co-expression modules by WGCNA. Among them, the module including CLEC4A, COTL1, EVI2B, FCER1G, GAPT, HCST, NCF2, PILRA, TLR8, and TYROBP had the highest correlation with AF. Four Hub genes (PILRA, NCF2, EVI2B, GAPT) were obtained further by LASSO analysis. The results suggested that the expression level of PILRA was significantly elevated in the rats with AF by qPCR, compared to the rats without AF. The results revealed that the infiltration of neutrophils, macrophages, monocytes, mast cells, immature B cells, myeloid-derived suppressor cell (MDSC), dendritic cell, and T cells and their partial subpopulations were closely related to AF by ssGSEA analysis, and PILRA was positively correlated with immature B cell, monocyte, macrophage, mast cell, dendritic cell, and T cells and their partial subpopulations by Spearman correlation analysis.

**Conclusions:**

PILRA was closely related to multiple types of immune cell infiltration, which may be associated with AF. PILRA may be a novel target of intervention for AF.

## 1. Introduction

Atrial fibrillation (AF) is the most common tachyarrhythmia (1), which increases the risk of heart failure and stroke, and is correlated with significantly increased mortality and morbidity (2, 3).

Currently, the atrial remolding including the structural and electrical remodeling is considered to play a crucial role in the pathogenesis of AF (4). The presence of inflammation in the heart or systemic circulation can predict the occurrence and recurrence of AF in the general population, as well as in patients after cardiac surgery, cardioversion, and catheter ablation. Moreover, increasing evidences indicate that there is a close correlation between inflammation and AF (5). Activation of NLRP3 inflammasome was observed in atrial cardiomyocytes of patients with paroxysmal and chronic AF, and the activation of NLRP3 inflammasome promoted AF by recruiting the immune cells and enhancing atrial structural and electrical remodeling (6). The infiltration of immune cells mediates the inflammatory response in cardiac tissue and provides substrates for AF-maintaining (7). However, the regulatory mechanism of the infiltration of immune cells in the atrial tissue of patients with AF is still not fully elucidated.

Weighted gene co-expression network analysis (WGCNA) and least absolute shrinkage selection operator (LASSO) regression analysis are important methods in bioinformatics. WGCNA can be used to find clusters of highly correlated genes, and summarize such clusters with the module eigengene or an intramodular hub gene, in order to relate modules to one another and to external sample traits (using eigengene network methodology), and to calculate module membership measures. It can improve stability of identification for candidate biomarkers or therapeutic targets (8). LASSO regression analysis is a shrinkage and variable selection method for linear regression models that can improve the selection and classification of relevant variables, compared to the traditional Cox regression and logistic regression (9). WGCNA and LASSO regression analysis could improve the accuracy of screening for disease Hub genes.

In this study, we explored the key differential genes of immune cells infiltration in AF using WGCNA and LASSO regression analysis simultaneously, aimed to provide new insights for mechanism of immune cells infiltration in AF, and to look for potential therapeutic targets for AF.

## 2. Materials and methods

### 2.1. Data collection

**Figure 1:**
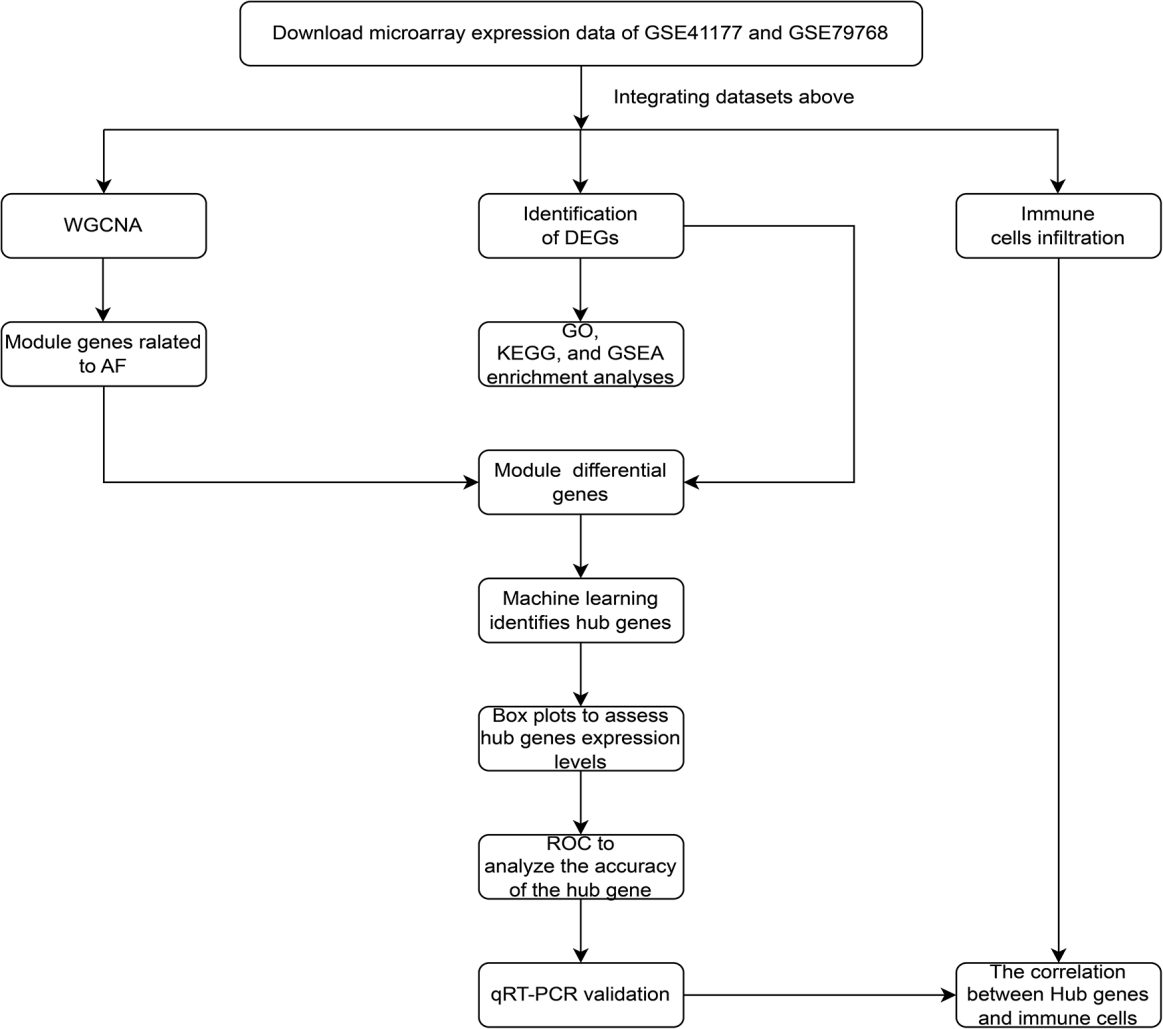
The study flowchart of data collection, analysis, processing, and experimental validation.

The study flowchart was shown in Figure 1. The datasets (GSE41177 and GSE79768) were obtained from the Gene Expression Omnibus database (GEO, http://www.ncbi.nlm.nih.gov/geo/), There are 14 AF samples and 12 sinus rhythm samples from 7 patients with AF and 6 patients with sinus rhythm in the dataset GSE79768 (GPL570 HG-U133_Plus_2 Affymetrix Human Genome U133 Plus 2.0 Array) (10), and 32 AF samples and 6 sinus rhythm samples from 16 patients with AF and 3 patients with sinus rhythm in the dataset GSE41177 (GPL570 HG-U133_Plus_2 Affymetrix Human Genome U133 Plus 2.0 Array) (11). Therefore, 46 AF samples and 18 sinus rhythm samples from 23 patients with AF and 9 patients with sinus rhythm were included in our analysis. Of note, left atrial tissue sample and right atrial tissue sample were respectively obtained from every patient in the dataset GSE79768, and tissue sample in left atrial appendage and tissue sample in left atrium-pulmonary vein junction were respectively obtained from every patient in the dataset GSE41177.

Subsequently, the normalization of the datasets was performed using the “SVA” package. We first read the genes information of two datasets to obtain each gene symbol. When different expression values were presented in a same gene, the mean values were calculated. Logarithmic transformation was performed for the non-logarithmic value of genes expression. Then, two datasets were combined and internally normalized. Finally, the normalization of the dataset was output.

The protocols of 2 studies analyzed in our study were conducted according to the Helsinki Declaration, and obtained the ethical approval. All patients enrolled in the 2 studies were given written informed consent (10, 11).

### 2.2. Acquisition of DEGs

The genes were analyzed by using “limma” package in R software, thresholded at |logFC| > 0.5 and adjusted *p* value <0.05. When different expression values were presented in a same gene, the mean values were calculated. When the genes expression values were 0, the genes were excluded. Then, the differential expression genes (DEGs) between samples with AF and sinus rhythm were obtained, when adjusted *p* values <0.05 correcting for the false discovery rate method (12). Finally, the DEGs were output and visualized by heatmap.

### 2.3. Enrichment analysis of DEGs

We then performed GO, KEGG, and GSEA enrichment analyses of DEGs by using “clusterProfiler and enrichplot” packages, and the immunological signature genome was used as a reference for GSEA. A *p* value <0.05 was considered statistically significant.

### 2.4. Genes co-expression network analysis

We constructed a weighted co-expression network using the WGCNA package to normalize the expression profiles of GSE41177 and GSE79768 batches. We checked the data by using “goodSampleGenes” and used the “pickSoftThreshold” function to obtain the appropriate soft threshold (*β*), then created a scale-free topology network while converting the matrix data into an adjacency matrix. After calculating module eigengene (ME) and merging similar modules in the clustering tree according to ME, we obtained a hierarchical clustering dendrogram. Gene significance (GS) and module significance (MS), which were used to measure genetic and clinical information, were calculated, and the correlations between modules and models were analyzed. Finally, we calculated the module membership (MM) for each gene to analyze the GS in the modules.

### 2.5. Screening of hub genes

We chose the LASSO regression algorithm to identify important variables as well as to improve the accuracy of prediction. The central genes with the lowest *p* value, high inter-module connectivity with |GS| > 0.50, and |MM| > 0.80, were firstly selected for screening of hub genes. Then, “Venn” package was used to intersect the central genes with DEGs. Finally, hub genes were obtained via “glmnet” analysis.

### 2.6. Validation of hub genes

We used the “ggpubr” package to construct box plots to assess hub genes expression levels between patients with AF and sinus rhythm, and plotted receiver operating characteristic curves (ROC) were used to analyze the accuracy of the hub gene for diagnosis by the “pROC” package.

### 2.7. Quantitative polymerase chain reaction

We constructed an animal AF model using the rats with transverse aortic constriction (TAC) (13–15). Meanwhile, the rats without TAC were used as control group. Eight rats were included in AF group, and 7 rats were included in control group. The temperature, appetite, and weight were detected in rats in each group in everyday, to exclude infectious diseases and heart failure. One month after TAC, the rats in each group were anesthetized and fixed supinely on an animal test bench. Then, vulnerability to AF of the rats in each group were measured by 3 cyclically transesophageal burst atrial pacing at a frequency of 1,500 stimuli/min for 30 s, with 5 min interval between cycles.

The experimental protocols were performed in accordance with the National Institutes of Health Guide for the Care and Use of Laboratory Animals, and were approved by the Ethics Committee of Guangxi Medical University Laboratory Animal Center (Nọ: 202207002).

The atrial tissue of rats in each group were collected after measurement of vulnerability to AF, and were snap frozen in liquid nitrogen for RNA isolation. Total RNA was extracted from the tissue using RNA extraction reagent (Servicebio, Wuhan, China). The concentration and purity of the extracted RNA were detected using an ultra-microspectrophotometer (Thermo Fisher Scientific, Massachusetts, USA). 1 μg of total RNA was reverse transcribed into cDNA using the Prime Script™ RT reagent Kit with gDNA Eraser (Takara, Tokoyo, Japan). The quantitative polymerase chain reaction (qRCR) procedure was carried out as follows: 95°C for 30 s, 95°C for 5 s, and 60°C for 30 s for 40 cycles. Melt-curve analysis was performed at 65–95°C. Glyceraldehyde-3-phosphate dehydrogenase (GAPDH) was used as the internal control for normalizing gene expression. The data obtained were calculated by the 2^–ΔΔCt^ method (16). The sequences of primers were shown in Table 1.

**Table 1:** The sequences of primers for qPCR.

Genes Name	Forward (5'–3')	Reverse (3'–5')
PILRA	GATTGACAGTGTTCCTCGGGTG	CTAGGTGCTGCTCCTGGTGA
NCF2	TAGGCTGTTCCGTCCAAATGA	AACCGTAGCCTTGCCCAGATA
GAPT	CAGCAGGCATAAAGACTACACGA	CAGTAGATTTCTGGCCTTTGCTT
EVI2B	AAAACCTATCAGACAAGCCCACA	GGCTCGTTGATCTGGGAATG
GAPDH	CTGGAGAAACCTGCCAAGTATG	GGTGGAAGAATGGGAGTTGCT

### 2.8. Immune cell infiltration

The ssGSEA algorithm was used to analyze the proportion of 28 types of immune cells in the immune infiltrative microenvironment of AF, the “vioplot” package was used to assess the immune cells infiltration between AF and control group, and Spearman correlation analysis was used to calculate the correlation between Hub genes expression and the proportion of immune cells infiltration.

### 2.9. Statistical analysis

qPCR data were analyzed using SPSS 23.0 software (SPSS Inc., Chicago, IL, USA). Two unpaired Student's *t*-test was performed to compare the difference between AF group and control group. All the results of qPCR in the study were visualized by graph pad Prism 8 (GraphPad Software, Inc., San Diego CA, USA). A *p* value <0.05 was considered statistically significant. All other statistical analyses were performed using R software version 4.1.0 (www.r-project.org/).

## 3. Results

### 3.1. Identification of DEGs

**Figure 2:**
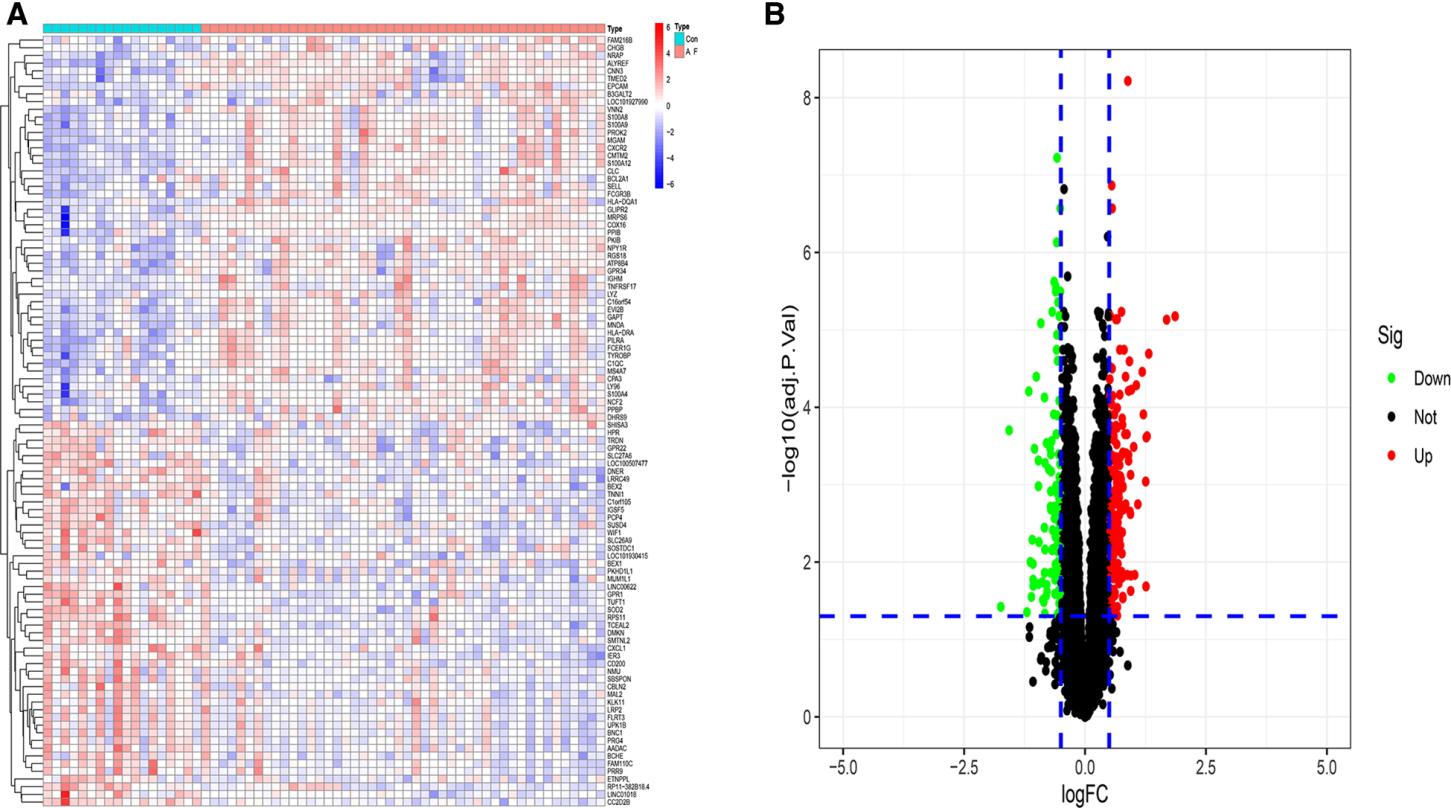
Identification differential expression genes of atrial fibrillation (**A**) heatmap of DGEs. (**B**) Volcano map of DGEs.

After the datasets were merged and normalized, a total of 298 DEGs were obtained. The results were shown in Figure 2.

### 3.2. Enrichment analysis of DEGs

**Figure 3:**
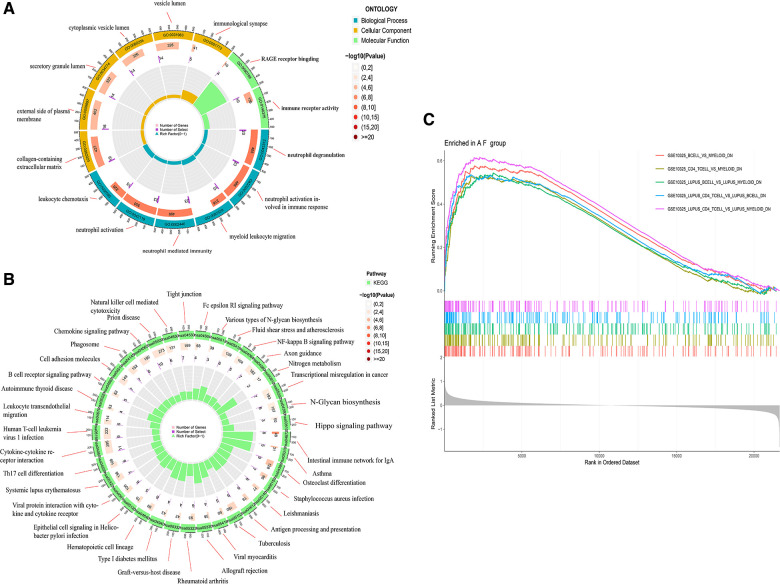
Enrichment analysis of DGEs in AF. (**A**) Gene ontology (GO) analysis of DEGs. (**B**) Kyoto encyclopedia of genes and genomes (KEGG) analysis of DEGs. (**C**) Gene set enrichment analysis (GSEA) of DEGs.

To understand the biological functions and signaling pathways associated with AF, we performed the differential enrichment analysis. GO enrichment analysis revealed that DEGs were associated with neutrophil degranulation, neutrophil activation involved in immune response, immune receptor activity, RAGE receptor binding, and collagen-containing extracellular matrix (Figure 3A). KEGG enrichment analysis showed that DEGs were associated with cytokine-cytokine receptor interactions, chemokine signaling pathways and B cell receptor signaling pathways, etc. (Figure 3B). The MsigDB database of the immune signature gene set was referenced for the GSEA to explore the potential mechanisms of immune function in AF. GSEA revealed that DEGs were significantly enriched in B cells, CD^4+^ T cells, CD^8+^ T cells (Figure 3C).

### 3.3. Construction of co-expression network

We used the WGCNA package to construct a gene co-expression network. After the samples were clustered to handle missing values and remove outliers, a scale-independent topological network was built (soft threshold *β* = 7, scale-free *R*^2^ = 0.83; slope = −1.59). A co-expression matrix was also constructed using a one-step method. Mixed cuts were made to construct a hierarchical clustering tree, and 10 gene modules were subsequently obtained by fusion of similar modules.

**Figure 4:**
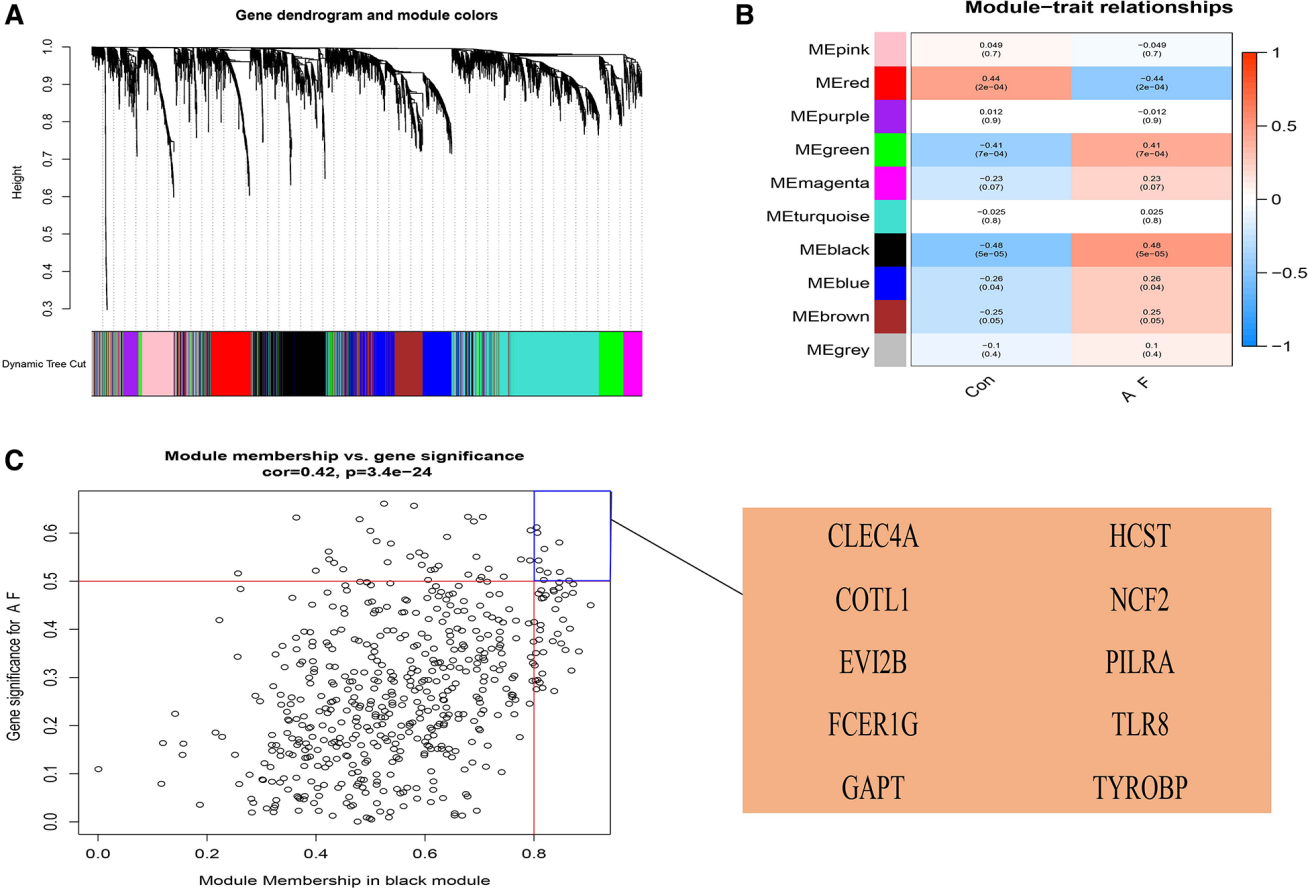
Construction of weighted gene co-expression network analysis (WGCNA) modules in AF. (**A**) The original and combined modules of tree diagram. (**B**) The black module has the highest correlation with AF in the heatmap of module-trait relationships. Red: positive correlations with AF; blue: negative correlations with AF. (**C**) The candidate genes contributing to AF in the black model shown in Scatter plot. When |GS| was more than 0.50 and |MM| was more than 0.80, the genes were chosen as the candidate genes. GS, gene significance; MM, module membership.

The correlation of the above modules with AF or sinus rhythm was clarified using heatmap. The results indicated that the module (including CLEC4A, COTL1, EVI2B, FCER1G, GAPT, HCST, NCF2, PILRA, TLR8, and TYROBP) had the highest correlation with AF (*ρ* = 0.48, *p* = 5 × 10^−5^) (Figure 4). Furthermore, there was a good correlation between GS and MM in this module (*ρ* = 0.42, *p *= 3.4 × 10^−24^). Therefore, we finally chose this module as the candidate module.

### 3.4. Screening of hub genes

**Figure 5:**
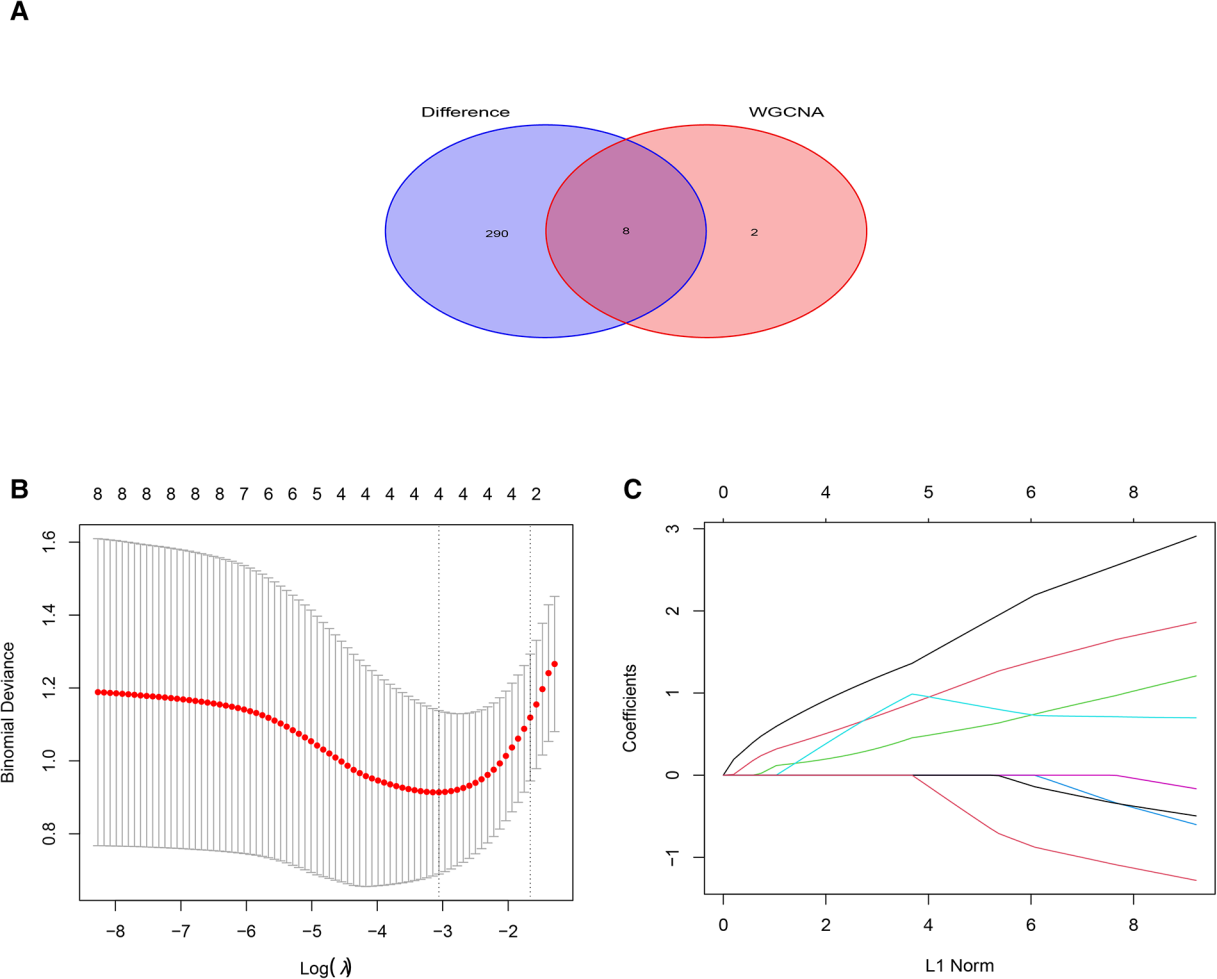
Least absolute shrinkage selection operator (LASSO) regression analysis of DGEs in AF. (**A**) Venn diagram of crossover genes between DEGs and candidate modules. (**B**) The trend of coefficient distribution of cross-validation. Four genes (PILRA, NCF2, EVI2B and GAPT) were obtained in the optimal *λ* value. (**C**) Distribution of pivotal genes in Lasso regression analysis.

Ten genes from the black module were obtained using |GS| > 0.50 and |MM| > 0.80 as a cutoff value, and analyzed using the LASSO algorithm. The 4 hub genes including PILRA, NCF2, EVI2B, and GAPT were obtained finally by LASSO regression analysis after intersecting with DEGs (Figure 5).

### 3.5. Expression levels and diagnostic effectiveness of hub genes

**Figure 6:**
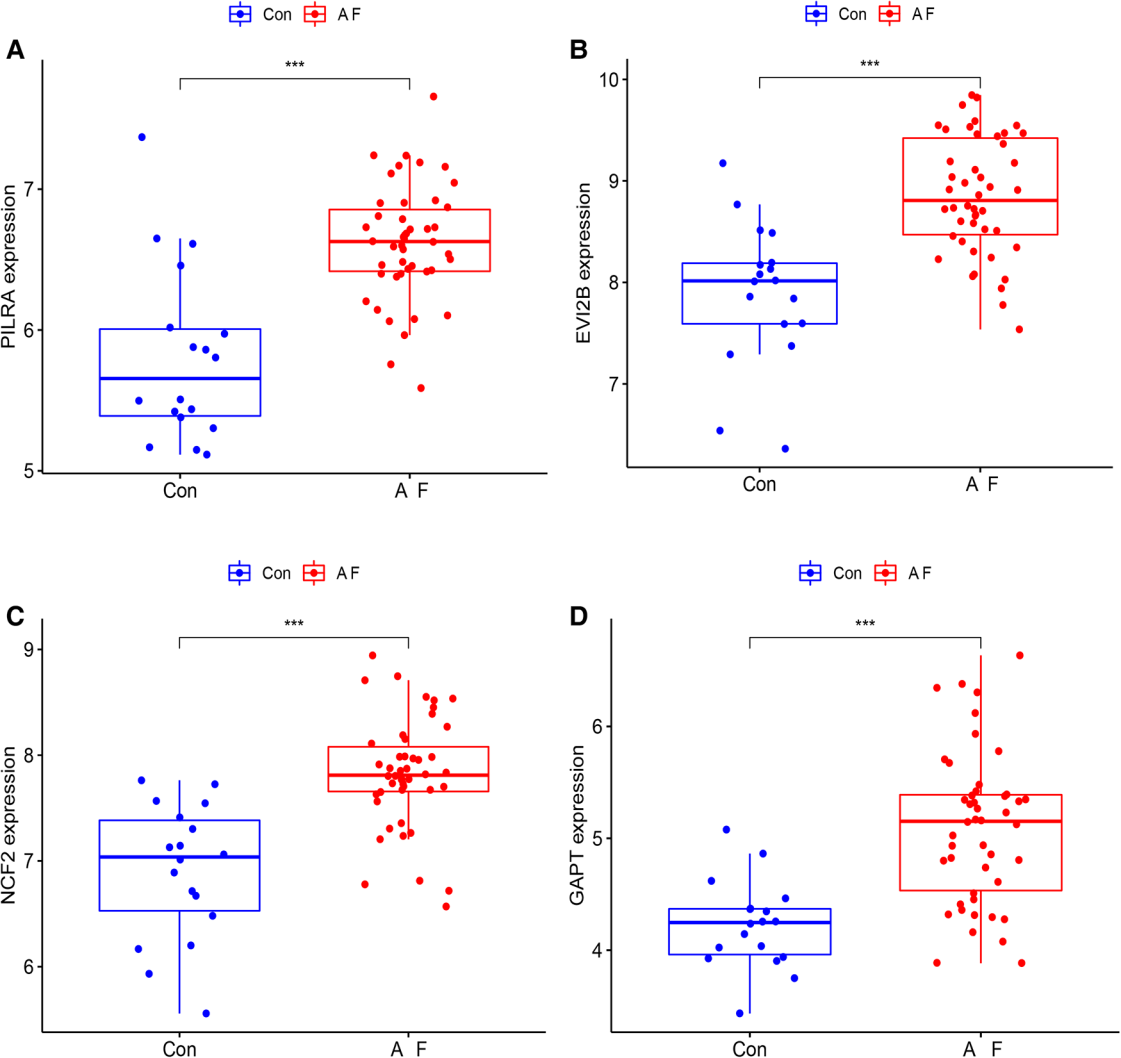
Elevated expression levels of PILRA, EVI2B, NCF2, and GAPT in atrial tissue of patients with AF from GSE41177 and GSE79768. (**A**) The expression of PILRA in patients with AF and patients with sinus rhythm. (**B**) The expression of EVI2B in patients with AF and patients with sinus rhythm. (**C**) The expression of NCF2 in patients with AF and patients with sinus rhythm. (**D**) The expression of GAPT in patients with AF and patients with sinus rhythm. Con: the patients with sinus rhythm. AF: the patients with AF. (***p < 0.001; **p < 0.01; *p < 0.05).

**Figure 7:**
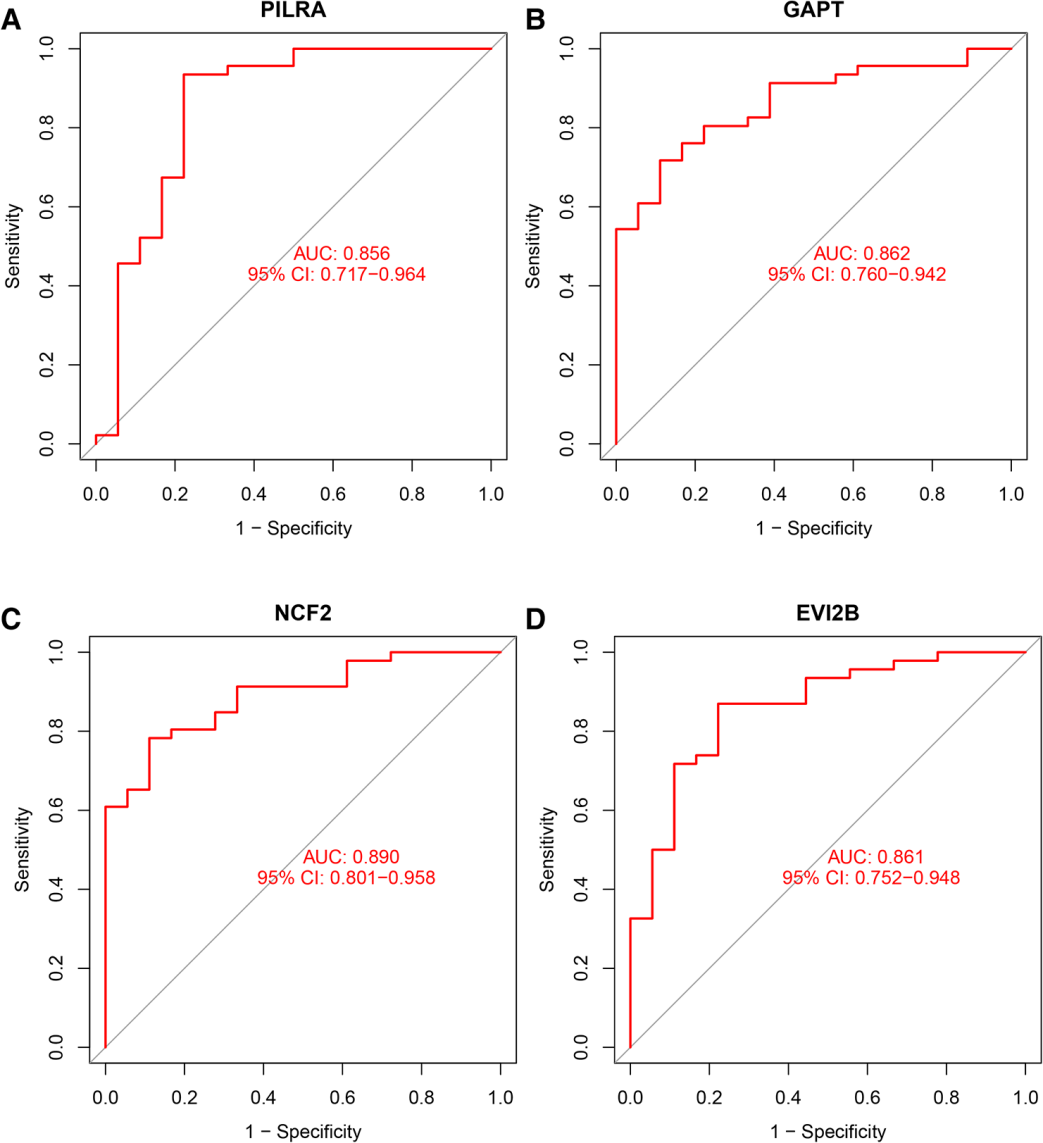
Area under curve (AUC) of the Hub genes for diagnosis of AF. (**A**) AUC value of PILRA. (**B**) AUC value of GAPT. (**C**) AUC value of NCF2. (**D**) AUC value of EVI2B.

We used box plots to verify the expression levels of the 4 Hub genes, the results presented in Figure 6 suggested that the expression of PILRA, NCF2, EVI2B, and GAPT were higher in atrial tissue of patients with AF, compared to those in atrial tissue of patients with SR. To verify whether the 4 hub genes have a good diagnostic value, we computed the values of the area under the curve (AUC) of the Hub genes. The AUC values of PILRA, NCF2, EVI2B, and GAPT were 0.856, 0.890, 0.861, and 0.862, respectively (Figure 7). These results indicated that these genes had a good diagnostic value for AF.

### 3.6. The expression levels of 4 hub genes in AF rat model

**Figure 8:**
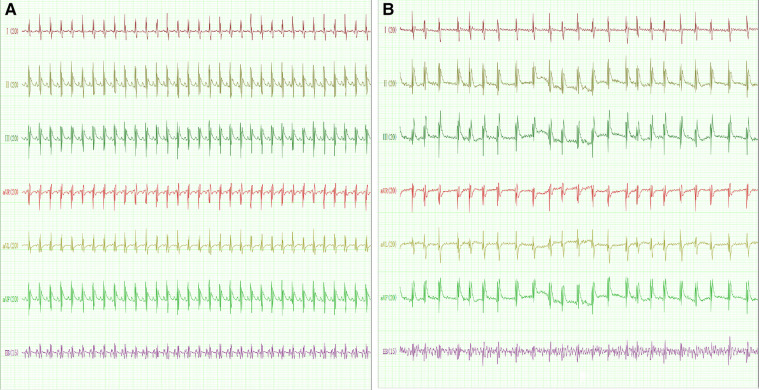
The electrocardiogram of rat with sinus rhythm (SR) or AF. (**A**) The representative electrocardiogram of rats with SR. *p* wave and regular R–R interval could be observed. (**B**) The representative electrocardiogram of rats with AF. *p* wave disappeared and was replaced by a disordered *f* wave with absolutely irregular R–R intervals. EB: transesophageal burst pacing.

**Figure 9:**
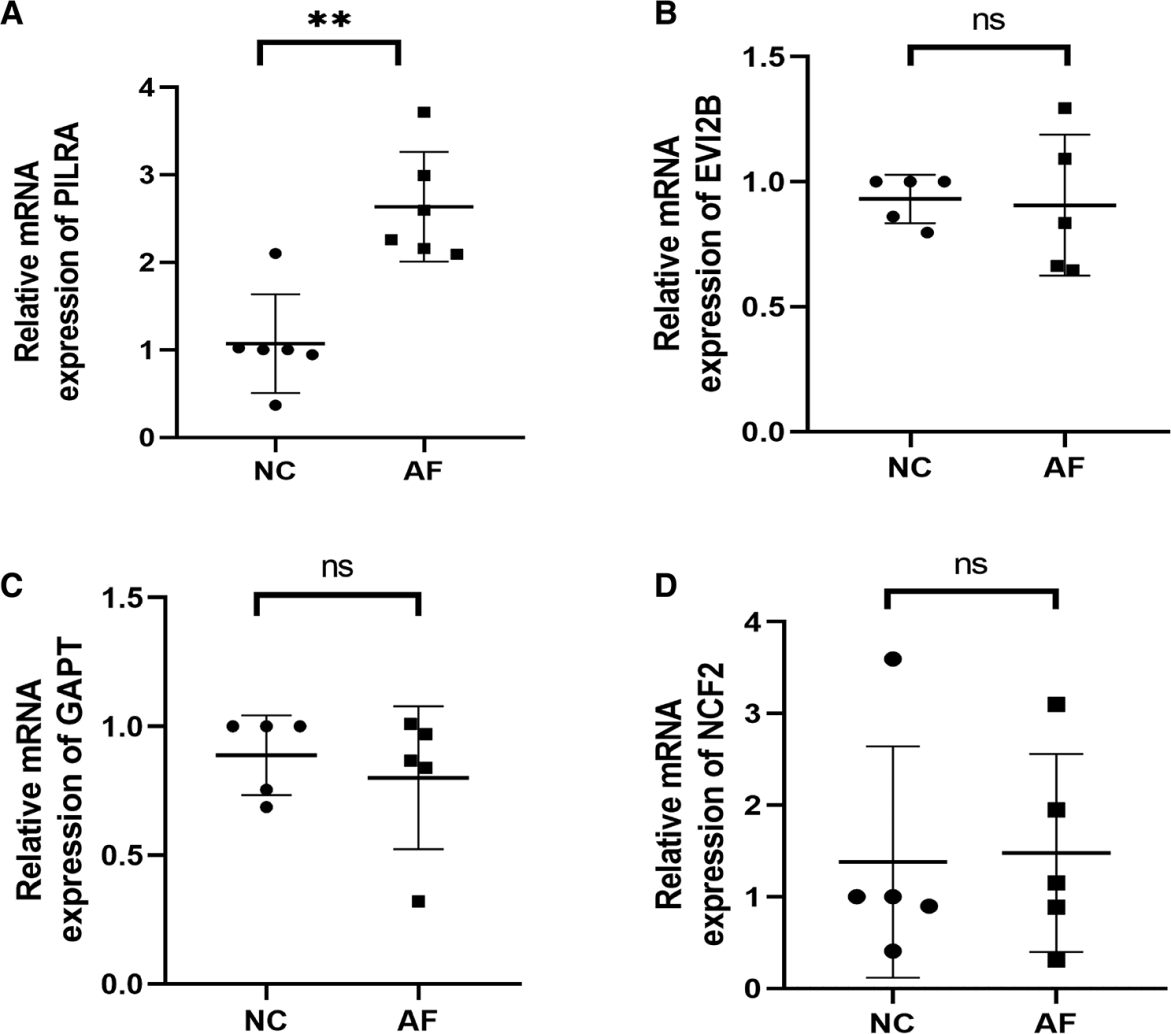
Relative mRNA expression levels of Hub genes. (**A**) PILRA expression level in rats with or without AF. (**B**) EVI2B expression level in rats with or without AF. (**C**) GAPT expression level in rats with or without AF. (**D**) NCF2 expression level in rats with or without AF. (**p < 0.01; ns, no significant difference).

The expression levels of 4 hub genes in rats with AF and sinus rhythm were measured by qPCR. There were 7 rats in Control group and 8 rats in AF group. Two rats in AF group with TAC were dead of heart failure. Finally, 7 rats in control group and 6 rats in AF group were performed transesophageal burst atrial pacing to measure the vulnerability of AF. The electrocardiograms of rats in control and AF groups were shown in Figure 8. The results have been shown in Table 2. One rat in Control group was dead after transesophageal burst pacing. The expression levels of PILRA were measured in 6 rats in each group, and expression of NCF2, EVI2B, and GAPT were measured in 5 rats in each group. The results shown in Figure 9 revealed that the expression level of PILRA was significantly elevated in rats with AF, compared to those in rats with SR, but the expression levels of NCF2, EVI2B, and GAPT were no obvious difference between the rats with AF and the rats with SR. These results further proved that PILRA may be a potential target for intervention of AF.

**Table 2:** The inducibility and duration of atrial fibrillation (AF) by transesophageal burst atrial pacing in control and AF group.

Group	Inducibility of AF	AF duration ≤45 s	AF duration ≥249 s	AF duration ≥500 s
Control (*n* = 7)	1 (14.3%)	1 (14.3%)	0	0
AF (*n* = 6)	6 (100%)	0	5 (83.3%)	1 (16.7%)

### 3.7. Assessment of immune cells infiltration

**Figure 10:**
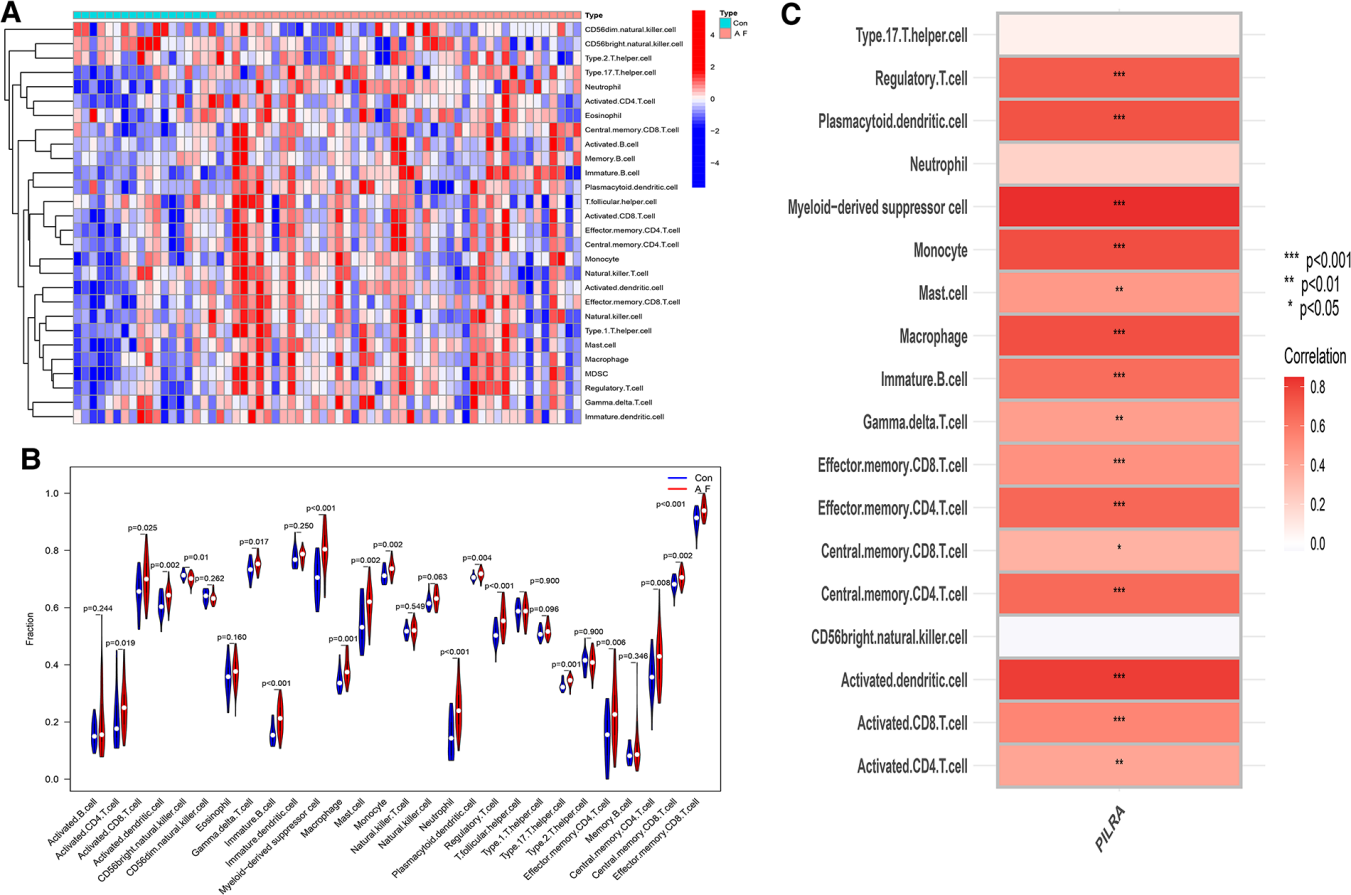
The association of PILRA with immune cell infiltration in AF. (**A**) Heatmap of immune cell infiltration in AF. (**B**)Violin plot showed the correlation between immune cells infiltration and AF. (**C**) The relationship between immune cells infiltration and PILRA.

We used the ssGSEA to explore the different types of immune cells between AF and SR, and evaluate their relationship. The distribution of immune cells in AF and SR, shown in Figures 10A,B revealed that immunity had a correlation with AF. Activated CD^4^ T cell (*p *= 0.019), Activated CD^8^ T cell (*p *= 0.024), Activated dendritic cell (*p *= 0.002), Gamm delta T cell (*p *= 0.016), Immature B cell (*p* < 0.001), myeloid-derived suppressor cell (MDSC) (*p* < 0.001), Macrophage (*p *= 0.001), Mast cell (*p *= 0.001), Monocyte (*p *= 0.002), Neutrophil (*p* < 0.001), Plasmacytoid dendritic cell (*p *= 0.003), Regulatory T cell (*p* < 0.001), Type 17 T helper cell (*p *= 0.001), Effector memory CD^4^ T cell (*p *= 0.005), Central memory CD^4^ T cell (*p *= 0.008), Central memory CD^8^ T cell (*p *= 0.001) and Effector Memory CD^8^ T cell (*p* < 0.001) were positively correlated with AF (Figure 10C).

The Spearman correlation analysis showed that PILRA was significantly and positively correlated with Immature B cell (*p* < 0.01), dendritic cell (*p* < 0.001), Myeloid–derived suppressor cell (*p* < 0.001), mast cell (*p* < 0.01), macrophage (*p* < 0.001), and T cells and their subpopulations (including Regulatory T cell, Effector memory CD^8^ T cell, Effector memory CD^4^ T cell, Central memory CD^4^ T cell, Activated CD^8^ T cell, Activated CD^4^ T cell (*p* < 0.001), Gamma delta T cell (*p* < 0.01), Central memory CD^8^ T cell (*p* < 0.05). These results suggested that PILRA may play a crucial role in regulation of immune cells infiltration that enhanced inflammatory response to promote AF, and PILRA may be a novel target for intervention of AF.

## 4. Discussion

In this study, we integrated two AF microarray datasets from GEO to identify the Hub genes associated with immune infiltration in AF. 298 DEGs were obtained after the 2 datasets were merged and normalized. The DEGs were significantly associated with adhesion and infiltration of immune cells, and immune response by GO enrichment analysis, KEGG enrichment analysis, and GSEA enrichment analysis, respectively. Then, the module that had the highest correlation with AF was obtained by WGCNA. Subsequently, the genes from the module were analyzed by LASSO regression analysis. The results highlighted that PILRA, NCF2, EVI2B, and GAPT may be the Hub genes. Furthermore, we detected the expression of 4 Hub genes by qPCR. We found that only expression level of PILRA was elevated in AF rat model, compared to those in sinus rhythm rat model, and PILRA was significantly and positively correlated with infiltration of multiple types of immune cells that were involved in AF. Taken together, our results suggested that PILRA may play a crucial role in regulation of immune cells infiltration that enhanced inflammatory response to promote AF, and PILRA may be a novel target for intervention of AF.

Interestingly, most of the DEGs were related to adhesion and infiltration of immune cells, and immune response by GO enrichment analysis, KEGG enrichment analysis, and GSEA enrichment analysis, respectively. This indicates that inflammation plays an important role in initiation and perpetuation of AF. KEGG enrichment analysis showed that DEGs were associated with cytokine-cytokine receptor interactions, chemokine signaling pathways, and B cell receptor signaling pathways. Cytokines are a class of proteins in inflammatory signaling pathway including chemokines, interleukins, and tumor necrosis factors-α (TNF-α) (17). TNF-α was associated with the pathogenesis of chronic AF, and increased levels of TNF-α and atrial fibrosis were often detected in AF patients with valvular heart disease (18). Systemic inflammation with elevated serum levels of interleukin 6, induced atrial electrical remodeling through the downregulation of cardiac connexins, and increased the risk of AF (19). Chemokines are a large family of small, inducible, secreted proteins that bind to G-protein–coupled receptors on target cells and have the ability to recruit leukocytes to sites of injury. A recent study showed that chemokine receptor CXCR-2 initiated AF by triggering monocyte mobilization in mice (20). Another study revealed that the chemokine signaling pathway, CXCL12/CXCR4 axis, enhanced atrial inflammation and fibrosis by recruiting CD^3+^ T lymphocytes and F4/80+ macrophages (21). In addition, it was also found that chemokine CXCL10 contributed to atrial inflammation and AF (22). All the above evidences uncovered that the DEGs may enhance inflammatory response by regulating the infiltration of immune cells to promote AF.

To look for the key genes correlated with AF, a gene co-expression network was constructed by WGCNA. The genes from the module, which had the highest correlation with AF by WGCNA, were further analyzed by LASSO regression analysis. Finally, 4 Hub genes including PILRA, NCF2, EVI2B, and GAPT were found. To prove the validity of 4 Hub genes, we measured their expression levels between rats with AF and rats with sinus rhythm using qPCR. The results showed that only expression of PILRA was elevated in AF, consisting with bioinformatics analysis.

We further analyzed the types of immune cells with increased infiltration in AF. We found increased infiltration of activated CD^4^ T cell, activated CD^8^ T cell, activated dendritic cell, gamma delta T cell, immature B cell, MDSC, macrophage, mast cell, monocyte, neutrophil, plasmacytoid dendritic cell, regulatory T cell, type 17 T helper cell, effector memory CD^4^ T cell, central memory CD^4^ T cell, central memory CD^8^ T cell, and effector memory CD^8^ T cell in AF.

In the atrium of human with AF, cellular inflammation, mainly consisting of functional cytotoxic T lymphocytes, was observed (23). The peripheral percentage of Th1 cells, the absolute number of Th17 cells, and the ratio of Th1/Treg were associated with a significantly higher risk of AF in patients with rheumatoid arthritis (24). CD^4+^CD^28null^ T cells are associated with the development of AF after elective cardiac surgery (25). Similarly, CD^8+^CD28^null^ T cells are associated with the development of AF after elective cardiac surgery (26). These pieces of evidence showed that T cells and their subpopulations play an important role in inflammatory response to promote AF. Our results are similar to these previous reports. However, the effects of B cells and their subpopulation on inflammatory response leading to AF are still unknown.

The recruitment of monocytes/macrophages and neutrophils is enhanced in the atrial tissue of patients with AF or in angiotension II–induced mouse AF model (27). Pressure overload induced mast cells infiltration and fibrosis in the atrium of mice and enhanced AF susceptibility following atrial burst stimulation (28). In addition, the infiltrated myeloid dendritic cells, migratory-active dendritic cells, and mature dendritic cells were significantly higher in left atrium of the patients with rheumatic heart disease than the patients without rheumatic heart disease, which was closely associated with inflammation and atrial remolding (29). In accordance with these results, our study uncovered that the infiltration of monocytes, macrophages, neutrophils, and dendritic cells was correlated with AF.

Moreover, PILRA was significantly and positively correlated with activated CD^4^ T cell, activated CD^8^ T cell, regulatory T cell, effector memory CD^4^ T cell, effector memory CD^8^ T cell, central memory CD^4^ T cell, central memory CD^8^ T cell, gamma delta T cell, immature B cell, monocyte, macrophage, mast cell, activated dendritic cell, and plasmacytoid dendritic cell. These results suggested that PILRA was an important regulator of infiltration of immune cells in AF.

PILRα encoded by PILRA was an inhibitory receptor containing immunoreceptor tyrosine-based inhibitory motifs (ITIMs), negatively regulated neutrophil infiltration during inflammation (30). It was also able to interact with CD^8α^ to maintain CD^8+^ T cell quiescence (31). In addition, PILRα can inhibit monocyte migration into tissue and differentiation into macrophages through regulating integrin signaling and inhibiting CD^99^–CD^99^ binding (32). We speculate that the elevated level of PILRA in AF may be a compensatory mechanism to protect against inflammatory injury by preventing the activation of CD^8+^ T cells and monocyte. However, this compensatory power may be not enough to reverse the inflammatory injury led by other immune cells. This is similar to activation of natriuretic peptide system in heart failure. The increased level of brain natriuretic peptide is observed in patients with heart failure, but increased level of endogenous brain natriuretic peptide is not enough to improve the symptom of patients with heart failure. Moreover, administration of exogenous recombinant human brain natriuretic peptide can markedly alleviate the symptoms of heart failure. Therefore, we think that PILRA may be a novel target of intervention for AF. To the best of our knowledge, it is the first study to report that PILRA is a potential regulator of immune cells infiltration in AF. However, the effects of PILRA on infiltration of T cell subsets except CD^8+^ T cell, B cell, mast cell, and dendritic cell are needed to clarify.

Our research has several limitations. Firstly, it was a bioinformatics analysis. Although 2 GEO datasets were merged, and WGCNA and LASSO regression analysis were used to obtain the Hub genes of AF, the exact roles of 4 Hub genes in AF are needed to clarify by vitro and vivo studies. Secondly, the results of qPCR showed that elevated level of PILRA, but no significant difference among the expression levels of NCF2, EVI2B, and GAPT between the rats with and without AF were observed. Our results from a small sample animal study need extensive samples of atrial tissue of human with and without AF for validation. Finally, the elevated level of PILRA in AF was preliminary determined by qPCR, and the correlation between PILRA and infiltration of immune cells was obtained by bioinformatics analysis. Gain- and loss-of-function of PILRA in animal are needed to investigate the effects on immune cells infiltration and AF susceptibility, to further validate our conclusion.

In conclusion, PILRA was closely related to multiple types of immune cells infiltration, which may be associated with AF. PILRA may be a novel target of intervention for AF.

## Data Availability

Publicly available datasets were analyzed in this study. This data can be found here: http://www.ncbi.nlm.nih.gov/geo/, GSE41177 and GSE79768
